# *Bacteroides fragilis* fucosidases facilitate growth and invasion of *Campylobacter jejuni* in the presence of mucins

**DOI:** 10.1111/cmi.13252

**Published:** 2020-09-21

**Authors:** Yvette M.C.A. Luijkx, Nancy M.C. Bleumink, Jianbing Jiang, Herman S. Overkleeft, Marc M.S.M. Wösten, Karin Strijbis, Tom Wennekes

**Affiliations:** ^1^ Department Chemical Biology and Drug Discovery, Utrecht Institute for Pharmaceutical Sciences and Bijvoet Center for Biomolecular Research Utrecht University Utrecht The Netherlands; ^2^ Department Biomolecular Health Sciences Utrecht University Utrecht The Netherlands; ^3^ Leiden institute of Chemistry Leiden University Leiden The Netherlands; ^4^ Health Science Center, School of Pharmacy Shenzhen University Shenzhen China

**Keywords:** *Bacteroides fragillis*, *Campylobacter jejuni*, fucosidase, l‐fucose, mucin, virulence

## Abstract

The enteropathogenic bacterium, *Campylobacter jejuni*, was considered to be non‐saccharolytic, but recently it emerged that l‐fucose plays a central role in *C. jejuni* virulence. Half of *C. jejuni* clinical isolates possess an operon for l‐fucose utilisation. In the intestinal tract, l‐fucose is abundantly available in mucin *O*‐linked glycan structures, but *C. jejuni* lacks a fucosidase enzyme essential to release the l‐fucose. We set out to determine how *C. jejuni* can gain access to these intestinal l‐fucosides. Growth of the *fuc* + *C. jejuni* strains, 129,108 and NCTC 11168, increased in the presence of l‐fucose while fucose permease knockout strains did not benefit from additional l‐fucose. With fucosidase assays and an activity‐based probe, we confirmed that *Bacteriodes fragilis*, an abundant member of the intestinal microbiota, secretes active fucosidases. In the presence of mucins, *C. jejuni* was dependent on *B. fragilis* fucosidase activity for increased growth. *Campylobacter jejuni* invaded Caco‐2 intestinal cells that express complex *O*‐linked glycan structures that contain l‐fucose. In infection experiments, *C. jejuni* was more invasive in the presence of *B. fragilis* and this increase is due to fucosidase activity. We conclude that *C. jejuni fuc* + strains are dependent on exogenous fucosidases for increased growth and invasion.

## 1 INTRODUCTION

Enteropathogenic bacteria that infect the human intestinal tract have the ability to proliferate and invade within the intestinal niche that is dominated by its mucus layer and associated O‐glycans (Johansson, 2012; Johansson, Sjövall, & Hansson, 2013). The intestinal mucus layer consists of two distinct layers that together form a physical barrier protecting the underlying epithelium against invasive pathogens and the residing intestinal microbiota (Corfield, 2018; Cornick, Tawiah, & Chadee, 2015; Johansson, Holmén Larsson, & Hansson, 2011). Under healthy conditions, the microbiota are associated with the outer mucus layer while the inner layer is relatively impenetrable by bacteria (Johansson et al., 2014). At the host–microbe interface, the highly glycosylated mucins provide the resident microbiota with nutrients that are primarily derived from its *O*‐glycans (Ferreyra, Ng, & Sonnenburg, 2014). Invading enteropathogenic bacteria have to compete for nutrients and space at this interface in order to proliferate and cause infection.

Mucin *O*‐linked glycans contribute 80% of the total dry weight of intestinal mucins and are made up of the monosaccharides fucose, galactose, *N*‐acetylgalactosamine, *N*‐acetylneuraminic acid (sialic acid) and *N*‐acetylglucosamine (Bergstrom & Xia, 2013; Thiele et al., 2015). Numerous bacterial glycosidases have been identified that liberate monosaccharides from the complex *O*‐glycan structure (Thiele et al., 2015, PMID:24270786). It has been predicted by bioinformatics that up to 40% of all commensal bacterial encode for carbohydrate‐degrading enzymes (glycosidases) and monosaccharide‐utilising enzymes (Josenhans, Müthing, Elling, Bartfeld, & Schmidt, 2020).

One bacterial phylum that is well‐equipped to degrade polysaccharides is the Bacteroidetes that make up almost half of the bacteria found in the intestine (Eckburg et al., 2005). Genomic and proteomic analyses of *Bacteroides thetaiotaomicron* demonstrated that this bacterium possesses over 280 glycosidases, of which 11% are located on the outer membrane or released extracellularly (Eckburg et al., 2005; Comstock & Kasper, 2006, http://www.cazy.org/b5118.html). Sialidases and fucosidases are the glycosidases that target the two major terminal epitopes found on mucin *O*‐linked glycans, sialic acid and fucose. In the human intestine, the density of the fucosylated *O*‐glycans decreases from ileum to colon while sialylated *O*‐glycans show the reversed pattern (Thiele et al., 2015). Both fucose and sialic acid are reported in the literature to be correlated with the ability of pathogenic bacteria to thrive within the gut (Li et al., 2019; Ng et al., 2013; Pacheco et al., 2012). Enteropathogenic bacteria need to compete for nutrients with resident commensals to gain a foothold in our gut and it is thus important to understand how they access mucin‐derived monosaccharides to establish infection.

The enteropathogen *Campylobacter jejuni* is a microaerophilic Gram‐negative, spiral‐shaped bacterium with bipolar flagella. This species is the leading cause of bacterial gastroenteritis in the developing world (Acheson & Allos, 2001; Kaakoush, Castaño‐Rodríguez, Mitchell, & Man, 2015; Silva et al., 2011). *Campylobacter jejuni* isolates were for a long time considered to be non‐saccharolytic, meaning that these bacteria do not metabolise sugars (Hofreuter, 2014; Stahl, Butcher, & Stintzi, 2012). However, both Muraoka and Zhang and Stahl et al., have reported that certain strains of *C. jejuni* have the ability to utilise l‐fucose (Muraoka & Zhang, 2011; Stahl et al., 2011). They showed that two commonly studied *C. jejuni* strains, NCTC 11168 and RM1221, have a growth benefit in the presence of l‐fucose. Previous studies have identified potential similarities between the l‐fucose breakdown pathway of *C. jejuni* and the plant pathogen *Xanthomonas campestris* (van der Hooft et al., 2018). A recent study elucidated the l‐fucose breakdown pathway in the *C. jejuni* NCTC 11168 *fuc* + strain by solving the structure of a putative dehydrogenase, FucX, that can reduce l‐fucose and d‐arabinose in vitro (Garber et al., 2020).

Besides the effects of l‐fucose breakdown for basic metabolism, l‐fucose utilisation has a broader impact on *C. jejuni* biology. Transcriptomics analysis of *C. jejuni fuc* + strains showed a large‐fold change in transcript abundancies upon addition of l‐fucose with 74 transcripts up‐regulated and another 52 down‐regulated (Stahl et al., 2011). For example, up‐regulation was seen for the immunoreactive *cstA* (*cj0917c*), a carbon starvation protein A homologue (Nielsen et al., 2012; Stahl et al., 2011). However, explanations for these up‐ and down‐ regulations are not immediately clear. Furthermore, a recent metabolomics study has demonstrated that *C. jejuni fuc* + strains have an adaptive metabolome that changes in the presence of l‐fucose (van der Hooft et al., 2018). Metabolites dependent on l‐fucose, such as thiazolidine‐containing metabolites, could be detected that demonstrate the activation of metabolic pathways generating bio‐active compounds in *C. jejuni* (van der Hooft et al., 2018).

The fucose operon is not conserved universally among *C. jejuni* strains, but its presence has been linked to hyper invasiveness in in vitro virulence and transposon mutagenesis (Fearnley et al., 2008; Javed et al., 2010). The capacity of *C. jejuni fuc* + strains to utilise l‐fucose correlates with colonisation and pathogenicity advantages in neonatal piglet model (Stahl et al., 2011). Interestingly, the *C. jejuni* genomes that have been sequenced, so far, lack fucosidases that would be necessary to release l‐fucose from host mucins. A recent publication verified a lack of *C. jejuni* fucosidases and showed increased growth of *C. jejuni fuc* + strains in the presence of fucosidases secreted by commensal bacteria (Garber et al., 2020). Complementary to this finding, we want to determine the effect of fucosidase activity of residing commensal bacteria on *C. jejuni fuc* + strains hyper invasiveness in the presence of mucins.

Fucosidases can be classified in two different glycoside hydrolase families. GH29 fucosidases have a retaining mechanism by which they form a temporary covalent bond with fucosyl residue, while members of the GH95 family employ an inverting mechanism. GH29 enzymes (EC 3.2.1.111 and EC 3.2.1.51) have a broad specificity to α1,2/3/4/6‐fucosidic linkages, whereas GH95 enzymes (EC 3.2.1.63) are specific to α1,2‐fucosidic linkages. A previous proteomics study on *B. fragilis* showed the presence of two putative secreted GH29 α‐l‐fucosidases (Elhenawy, Debelyy, & Feldman, 2014). In our study, we utilise an activity‐based probe (ABP) that mimics a α‐l‐fucosyl residue and can detect catalytically active GH29 fucosidases (Jiang et al., 2015).

To investigate the dependence of *C. jejuni* on exogenous fucosidases activity and its implications for growth and virulence, we took an interdisciplinary approach by combining microbiology with an activity‐based probe (ABP), competitive fucosidase inhibitors and infection assays. With the use of the ABP, we were capable of visualising active GH29 fucosidases secreted by *B. fragilis*. Using this diverse approach, we demonstrated that fucosidases, secreted by commensal *Bacteroides fragilis*, increased growth and invasion of the *C. jejuni fuc* + strain in the presence of mucin *O*‐linked glycans. Our data shed light on nutrient competition in the intestinal tract and the contribution of commensal glycosidases to the virulence of enteropathogens.

## 2 RESULTS

### 2.1 The hyperinvasive *C. jejuni* 108 strain contains the genomic island required for fucose utilisation

**FIGURE 1:**
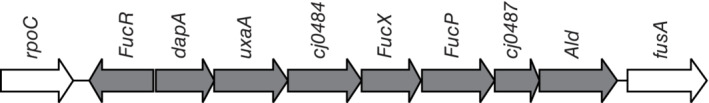
Schematic representation of the *fuc* + operon as it appears in *Campylobacter jejuni* NCTC 11168 and 1,219,108

The *fuc* + operon, containing genes, *cj0480c* to *cj0490*, has been identified in *C. jejuni* NCTC 11168 and RM1221 to be required for l‐fucose utilisation (Stahl et al., 2011). We sequenced the hyperinvasive *C. jejuni* 129,108 (108) strain and identified 11 genes to be homologous to the *cj0480*‐*cj0490* gene cluster of NCTC 11168 with a sequence similarity of 98.91%. Figure 1 shows the schematic representation of the *fuc* + operon. The genes encoded by the *C. jejuni fuc* + operon are predicted to include a transcriptional regulator (FucR), a synthase (dapA), a dehydratase (uxaA'), two major facilitator superfamily transporters (Cj0484 and FucP), two dehydrogenases (FucX and Cj0489), a hydrolase (Cj0487) and a mutarotase (Cj0488) (Garber et al., 2020; Stahl et al., 2011). Cj0486 is homologous to fucose permeases found in other bacteria and was previously shown to be an essential component of the active l‐fucose assimilation pathway in *C. jejuni* NCTC 11168 (Stahl et al., 2011). The predicted Cj0486 gene product in *C. jejuni* 108 is 99% identical to its NCTC 11168 homologue. Based on its sequence, we predict that the hyperinvasive *C. jejuni* 108 is a *fuc* + strain that has the ability to scavenge and metabolise l‐fucose.

### 2.2 l‐fucose increases growth of *C. jejuni* 108

**FIGURE 2:**
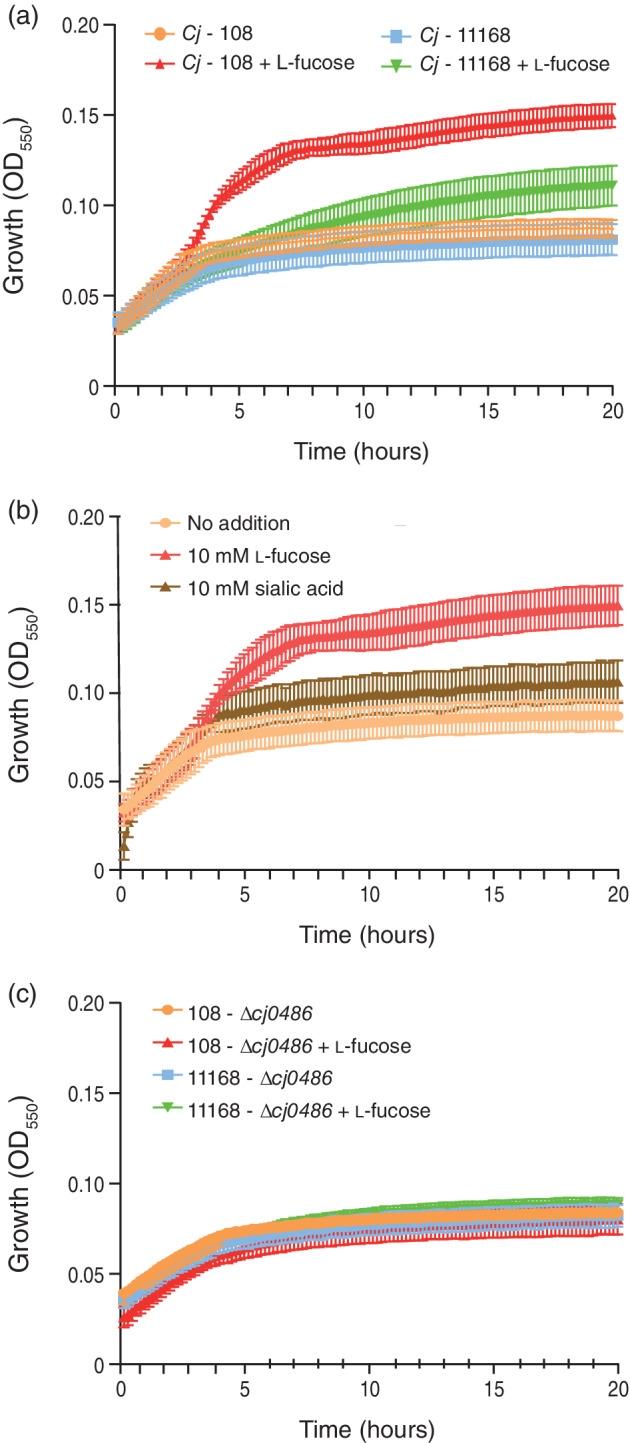
Growth of *Campylobacter jejuni* is enhanced in the presence of l‐fucose. Growth curves in DMEM of (a) *C. jejuni* strains, NCTC 11168 and 108, supplemented with either l‐fucose or sialic acid, (b) *C. jejuni* supplemented with 10 mM sialic acid or 10 mM l‐fucose, (c) *C. jejuni* NCTC 11168 and 108 Δcj0486 mutants with and without 10 mM l‐fucose. All experiments were performed three times, error bars show SEM

We next investigated the effect of l‐fucose on growth of the *C. jejuni* 108 strain. Strains 108 and NCTC 11168 were grown in DMEM with and without l‐fucose. In the presence of l‐fucose, both strains reached a higher final optical density, but the growth increase was most pronounced in the 108 strain (Figure 2a). In contrast with l‐fucose, no significant increase in growth of *C. jejuni* 108 was seen in the presence of sialic acid (Figure 2b). We generated deletion strains for the fucose permease cj0486 for both the 11,168 and 108 strains and tested their ability to grow on l‐fucose. The growth of the mutant strains was similar with or without addition of l‐fucose, indicating that both mutants lost their ability to utilise l‐fucose (Figure 2c). These results demonstrate that the hyperinvasive *C. jejuni* 108 strain contains a pathway for the uptake and metabolism of l‐fucose and that l‐fucose confers a growth benefit.

### 2.3 Detection of fucosidase activity of commensal *Bacteroides fragilis* using chemical tools

**FIGURE 3:**
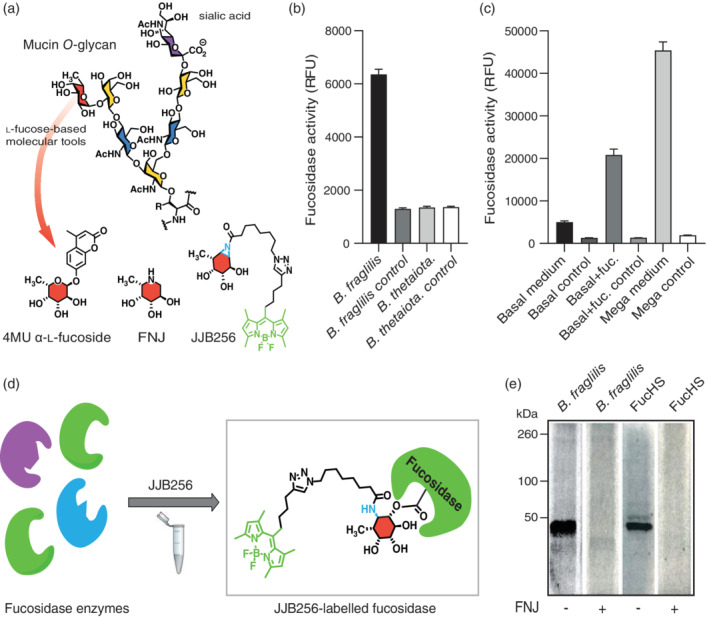
*Bacteroides fragilis* secretes active GH29 fucosidases. (a) Mucin *O*‐glycan structure and overview of the l‐fucose‐based molecular tools used in this study. 4MU‐fuc as fluorogenic substrate, reversible inhibitor FNJ, activity‐based probe JJB256 for in‐gel visualisation and quantification. Fucose (red), sialic acid (purple), *N*‐acetylgalactosamine (yellow), *N*‐acetylglucosamine (blue). Fucosidase activity as determined by 4‐methylumbelliferyl‐α‐l‐fucopyranoside (4MU α‐l‐fucopyranoside) assay in (b) supernatant fraction of *B. fragilis* and *B. thetaiotaomicron* grown on basal medium, negative controls show the background level of the heat‐inactivated samples, (c) *Bacteroides fragilis* supernatant after growth in basal medium, basal medium with 10 mM l‐fucose or the rich Mega medium. Fucosidase activity was expressed in relative fluorescence units (RFU) and negative controls show the background level of the heat‐inactivated samples. All experiments were performed three times, error bars show SEM. (d) Schematic representation of interaction of the JJB256 activity‐based probe (ABP) with a target GH29 fucosidase from a complex mixture of enzymes. (e) Detection of fucosidase activity with ABP JJB256 on FucHS and supernatant of *B. fragilis* by in‐gel fluorescence. As a negative control l‐fuconojirimycin (FNJ) was taken along

Our results demonstrate that *C. jejuni fuc* + strains can utilise the monosaccharide l‐fucose, but the release of l‐fucose from complex mucin *O*‐linked glycans requires extracellular fucosidase activity (mucin *O*‐glycan Figure 3a). We hypothesize that *C. jejuni fuc* + strains are dependent on fucosidase activity of residing commensals such as *Bacteroides* species. We selected *Bacteroides fragilis* and *Bacteroides thetaiotaomicron* for our experiments to induce extracellular fucosidases and detect their activity with a set of molecular tools (the structures of the tools used in our assays are depicted in Figure 3a). *Bacteroides fragilis* and *B. thetaiotaomicron* were grown anaerobically in basal medium (TYG) and activity of secreted fucosidases was measured by the breakdown of the fluorogenic substrate 4‐methylumbelliferyl‐α‐l‐fucopyranoside. Fucosidase activity was detectable in secreted fractions of *B. fragilis* grown under these conditions, but not in the supernatants of *B. thetaiotaomicron* or heat‐inactivated supernatant of *B. fragilis* (Figure 3b). Next, we determined if addition of l‐fucose or growth in Mega medium, which is specifically optimised for growth of intestinal anaerobic bacteria, would increase secreted fucosidase activity of *B. fragilis*. Addition of l‐fucose and growth in Mega medium enhanced secreted fucosidase activity (Figure 3c), which is in line with previous observations (Elhenawy et al., 2014).

In a previous study, proteomics analysis showed the presence of secreted GH29 fucosidases in this specific *B. fragilis* strain (Elhenawy et al., 2014). To confirm the nature of the fucosidase enzymes secreted by *B. fragilis* in our experimental set‐up, we used an activity‐based fucosidase probe (JJB256) that was previously synthesised and applied by the Overkleeft group (Jiang et al., 2015). When JJB256 is bound by a catalytically active GH29 fucosidase its reactive warhead (aziridine, blue, Figure 3d) will react to form a stable covalent bond within the fucosidase active site. The fluorophore that is attached to JJB256 (green, Figure 3d) allows for visualisation of the labelled fucosidases by in‐gel fluorescence (Figure 3e). We performed labeling experiments with JJB256 and found that it efficiently labelled the control *Homo sapiens* GH29 fucosidase (FucHS). In addition, we could detect positive labeling of fucosidases in the supernatant fraction of *B. fragilis* (Figure 3e). The prominent band around 50 kDa is in agreement with the predicted molecular weight of *B. fragilis* secreted fucosidases in the CAZy database (http://www.cazy.org/GH29.html). Pre‐incubation with 100 μM of the competitive fucosidase inhibitor l‐fuconojirimycin (FNJ) blocked labelling of the major 50 kDa bands supporting the presence of active GH29 fucosidases in the FucHS and *B. fragilis* supernatant fractions. We conclude that *Bacteroides fragilis* secretes active fucosidases of the GH29 family during anaerobic growth in Mega medium.

### 2.4 *Campylobacter jejuni* is dependent on *B. fragilis* fucosidases for growth on mucin

**FIGURE 4:**
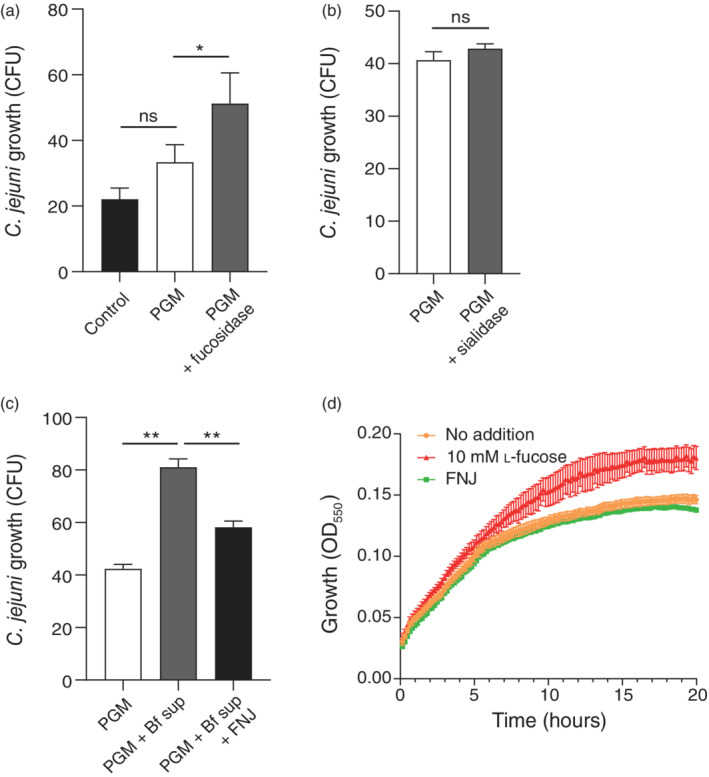
*Campylobacter jejuni* 108 requires exogenous fucosidases for growth on mucin. Growth of *C. jejuni* 108 on pig gastric mucin (PGM) without or with (a) recombinant fucosidase FucHS, (b) recombinant sialidase, (c) *B. fragilis* supernatant in the presence of 100 μM competitive fucosidase inhibitor FNJ. Data shown in bar graphs represent means ± SEM (*n* = 3 independent experiments); * *p* < .05; ** *p* < .01. (d) Growth curve of *C. jejuni* 108 in DMEM medium supplemented with 10 mM l‐fucose or 100 μM FNJ. Experiments were performed three times, error bars show SEM

We next investigated if *C. jejuni* 108 can benefit from exogenous fucosidase activity for growth on mucin *O*‐linked glycans. Porcine gastric mucin (PGM) was pretreated with or without purified fucosidase FucHS, and subsequently *C. jejuni* 108 was added and incubated for 24 hours. Pretreatment of the mucin with the fucosidase enzyme resulted in a significant increase in *C. jejuni* colony forming units (CFUs) compared to non‐treated PGM (Figure 4a). Pretreatment of PGM with sialidase did not confer a significant growth benefit for *C. jejuni* 108 (Figure 4b), which is in line with our earlier observations in the growth assays with sialic acid.

Next, we set out to investigate the effects of the more complex *B. fragilis* supernatant. We normalised fucosidase activity in the supernatant fraction to the previously used FucHS activity by comparing their in‐gel fluorescent signals with probe JJB256 using ImageJ software. PGM was pre‐treated with the concentrated *B. fragilis* supernatant fraction and added to the *C. jejuni* 108 culture. *Campylobacter jejuni* growth was significantly increased in the presence of the pre‐treated PGM compared to the untreated PGM (Figure 4c). Addition of 100 μM FNJ to the *B. fragilis* supernatant fraction resulted in a significant decrease in *C. jejuni* growth on PGM, demonstrating that the increase in *C. jejuni* growth is due to *B. fragilis* fucosidase activity (Figure 4d). As a control, we also investigated the effect of FNJ on the growth of *C. jejuni* 108. Growth curves of *C. jejuni* 108 were similar in the presence and absence of FNJ, indicating that this inhibitor does not directly impact growth (Figure 4d). Together, these results demonstrate that for growth on glycosylated mucin, *C. jejuni* 108 is dependent on secreted fucosidases from other species.

### 2.5 Effects of fucosidase activity on *C. jejuni* 108 invasion into intestinal epithelial cells

**FIGURE 5:**
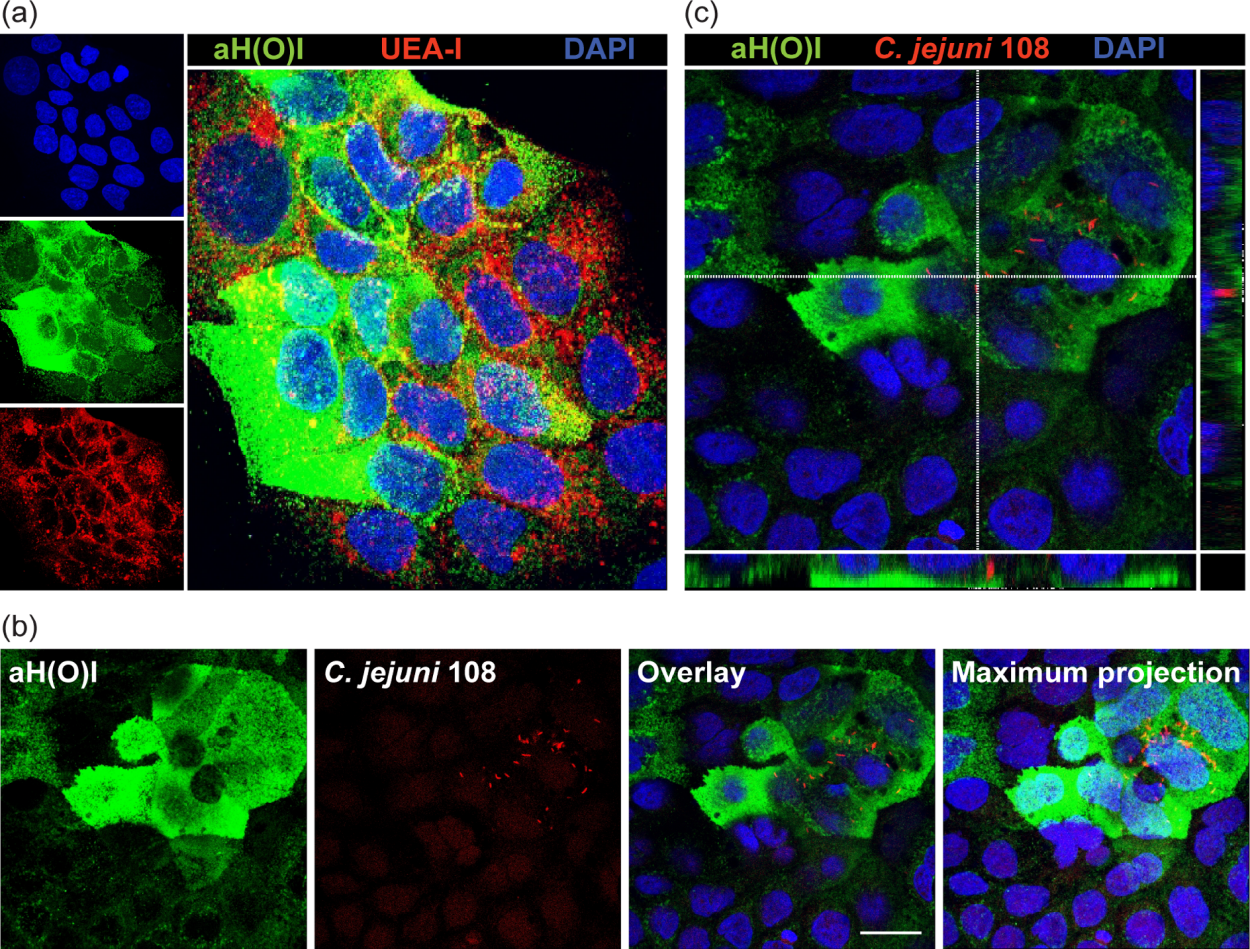
Presence of l‐fucosides on the apical surface of Caco‐2 epithelial cells correlates with *Campylobacter jejuni* 108 invasion. (a) Immunofluorescence confocal microscopy image of confluent Caco‐2 cells stained for l‐fucosides with UEA‐I lectin (α1,2 fucose H‐type 2, red), or aH(O)I antibody (α1,2 fucose H‐type 1, green) and nuclei (DAPI, blue). (b, c) Immunofluorescence confocal microscopy image of confluent Caco‐2 cells infected with *C. jejuni* 108 (mCherry, red) stained with aH(O)I antibody for α1,2 fucose H‐type 1 (green) and nuclei (DAPI, blue) demonstrating invasion of *C. jejuni* into Caco‐2 cells with apical l‐fucose. White scale bars represent 20 μm

Fucosylated mucins are also expressed on the apical surface of intestinal epithelial cells and could be possible targets for *C. jejuni* adhesion and invasion as previously suggested (Amano & Oshima, 1999; Ruiz‐Palacios, Cervantes, Ramos, Chavez‐Munguia, & Newburg, 2003). We investigated the presence of fucosylated mucins on the surface of the intestinal epithelial Caco‐2 cells after 5 days of differentiation. Immunofluorescence with UEA‐I lectin (H type 2) and the αH(O)I antibody (H type 1) showed the presence of H type 2 fucosylated structures evenly distributed over the cell surface and membrane‐bound mucin associated H‐type 1 fucosylation as patches (Figure 5a).

Microaerophilic growth conditions are optimal for *C. jejuni* and the importance of mimicking in vivo conditions for *C. jejuni* has previously been demonstrated (Mills et al., 2012). Therefore, we performed infection assays under microaerophilic conditions that are favourable to *C. jejuni* and more representative of the intestinal epithelial interface. A *C. jejuni* 108 strain, expressing mCherry, was used to allow imaging of invasion by confocal microscopy. Confluent Caco‐2 cells were differentiated for 5 days and infected with *C. jejuni* 108 for 3.5 h under microaerophilic conditions. We observed that *C. jejuni* 108 predominantly invaded l‐fucose positive cells (Figure 5b). Multiple Z‐stacks of the image were collected, and an orthogonal view showed apical fucosylated mucins with *C. jejuni* 108 located underneath (Figure 5c).

**FIGURE 6:**
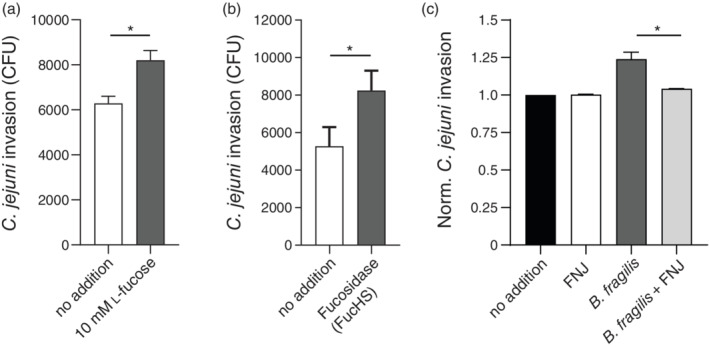
Increased invasion of *Campylobacter jejuni*108 into intestinal epithelial cells in the presence of l‐fucose. *Campylobacter jejuni* 108 gentamicin survival assays in Caco‐2 epithelial cells performed under microaerophilic conditions. Quantification of *C. jejuni* 108 invasion into Caco‐2 cells without or with 10 mM l‐fucose (a) and with or without fucosidase FucHS (b). *Bacteroides fragilis* in the presence or absence of the fucosidase inhibitor FNJ (c). Data shown in bar graphs represent means ± SEM (*n* = 3 independent experiments); * *p* < .05

To quantify invasion of *C. jejuni* into intestinal cells under different conditions, we performed gentamicin protection assays with Caco‐2 cells. Addition of 10 mM l‐fucose significantly increased the number of intracellular *C. jejuni* as measured by CFUs after lysis of the Caco‐2 cells (Figure 6a). This increased invasion might be, in part, attributed to the growth‐stimulating effect of l‐fucose on *C. jejuni* 108 that we observed previously. Next, we added FucHS during the infection assay to release l‐fucose from the mucin *O*‐glycans on the Caco‐2 cell surface. Addition of exogenous fucosidases resulted in a significant increase in intercellular *C. jejuni* 108 (Figure 6b). Based on these results, we conclude that free extracellular fucose increases *C. jejuni* invasion into intestinal epithelial cells.

As established above, *C. jejuni* benefits from fucosidase secreted by *B. fragilis* in the presence of fucosylated mucins. However, during infection in vivo, *Bacteroides* species also compete for the liberated l‐fucose (Hooper, Xu, Falk, Midtvedt, & Gordon, 1999). Therefore, our final experiment was to determine the effect of live *B. fragilis* bacteria on *C. jejuni* 108 invasion. *Bacteroides fragilis*, grown anaerobically in Mega medium, was added during the infection assay with Caco‐2 cells at a MOI 1:10 (*B. fragilis*: *C. jejuni 108*). To determine the impact of fucosidases on *C. jejuni* invasion, we conducted the experiment in the presence or absence of the competitive fucosidase inhibitor, FNJ. The presence of *B. fragilis* boosted *C. jejuni* 108 invasion and this effect was abolished in the presence of the competitive inhibitor, FNJ (Figure 6c). We conclude that the increased invasion of *C. jejuni* 108 was caused by fucosidase activity present during the co‐culture with *B. fragilis*. Furthermore, a previous study that reported on proteomics analysis of this *B. fragilis* strain showed only secreted GH29 fucosidases activity and no cell membrane bound fucosidases (Elhenawy et al., 2014). Taken together, these results support our hypothesis that locally liberated l‐fucose by secreted fucosidases from other species can increase growth and invasion of *fuc* + *C. jejuni* strains at the intestinal epithelial interface.

## 3 DISCUSSION

**FIGURE 7:**
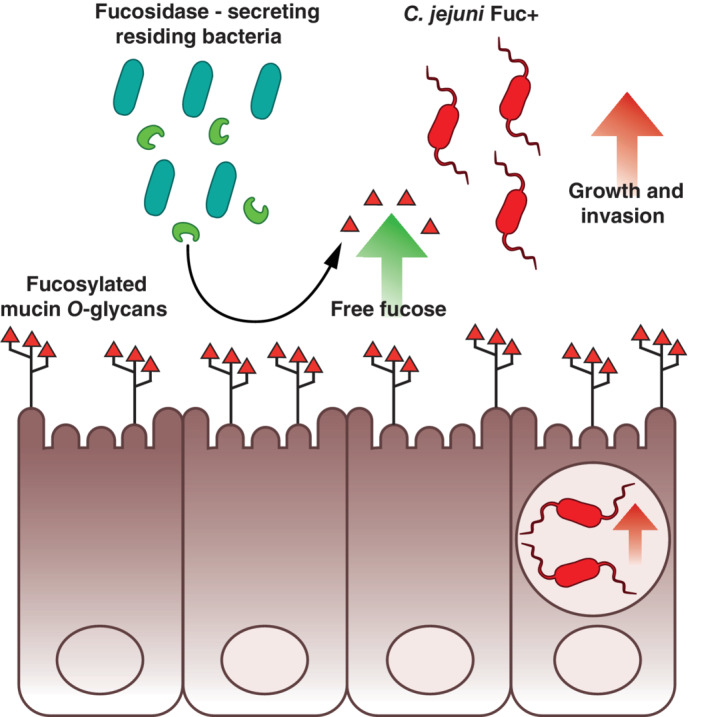
Schematic model of *Bacteroides fragilis* fucosidases enhancing growth and invasion of *Campylobacter jejuni fuc* + strains. Fucosylated mucin *O*‐glycans (red triangles) are expressed on the apical surface of intestinal epithelial cells. Residing bacteria, such as *Bacteroides fragilis*, secrete fucosidases (green), thereby liberating l‐fucose from the mucin *O*‐glycans. Our data show that l‐fucose and exogenous fucosidases stimulate increased growth and invasion of *C. jejuni fuc* + strains. We hypothesize that *C. jejuni fuc* + strains scavenge and metabolise l‐fucose and alter their invasive properties

When *C. jejuni* invades the mucosal lining of the intestinal epithelium it has to compete with residing intestinal microbiota for nutrients and space (Lee, O'Rourke, Barrington, & Trust, 1986). Although *C. jejuni* prefers growth on amino acids, some hyperinvasive strains possess the ability to metabolise l‐fucose (Fearnley et al., 2008; Javed et al., 2010), which is an abundant terminal component of mucin *O*‐glycans that cover the intestinal epithelium. The ability of *C. jejuni fuc* + strains to metabolise l‐fucose confers a competitive advantage in infection models (Stahl et al., 2011). However, as *C. jejuni* lacks endogenous fucosidases, it does not have the capacity to release l‐fucose from mucin *O*‐glycans. Our results here show that secreted fucosidases of a commensal, *Bacteroides fragilis*, facilitate enhanced growth of the hyperinvasive *C. jejuni fuc* + strain 108 on glycosylated mucins (Figure 7). Furthermore, we used activity‐based protein profiling (ABPP) and chemical competitive inhibitors to demonstrate the crucial contribution of these exogenous fucosidases to the increased invasion by *C. jejuni* 108 into intestinal epithelial cells. Our findings complement two recent publications on the topic of *C. jejuni fuc* + strains that investigated nutrient scavenging by *C. jejuni fuc +* strains and demonstrated how the presence of glycoproteins in human milk affects the selection of these specific strains (Bian et al., 2020; Garber et al., 2020). Furthermore, it is interesting to speculate that our data are in line with a previous finding that individuals with higher proportions of *Bacteroides* species are more susceptible to *C. jejuni* infections (Dicksved, Ellström, Engstrand, & Rautelin, 2014).

*Bacteroides* species are known to tightly regulate the secretion of their extracellular fucosidases, which levels often appear lower in vitro compared to in vivo experiments (Sonnenburg et al., 2005). ABPP is an established and powerful technique to label and detect catalytically active enzymes in their native environment (Cravatt, Wright, & Kozarich, 2008). Our successful application of chemical probe, JJB256, to label previously putative retaining GH29 α‐l‐fucosidases in the secretome of *B. fragilis* highlights the possible further application of this probe and potential future derivatives (Jiang et al., 2015). One such application could be screens for secreted GH29 fucosidases in both microbiota and pathogens.

The l‐fucose cross‐feeding that we observe between the commensal *Bacteroides fragillis*, and invading pathogen *Campylobacter jejuni* is a strategy encountered more often among enteropathogenic bacteria. The virulence genes of Enterohemorrhagic *E. coli* (EHEC) are regulated by the fucose‐activated FusKR signalling pathway. When EHEC is grown on mucins in the presence of *Bacteroides*, virulence genes are upregulated in a FusKR‐dependent manner. These results suggest that EHEC uses l‐fucose, liberated by *Bacteroides* fucosidases, to modulate its pathogenicity (Pacheco et al., 2012). This strategy is not limited to l‐fucose, as two other enteric pathogens, *Salmonella* Typhimurium and *Clostridium difficile*, have both been shown to use sialic acids that were liberated by sialidases expressed by microbiota (Ng et al., 2013). *Salmonella* Typhimurium shows a significant upregulation of genes involved in the sialic acid catabolism pathway when infecting mice that contain *Bacteroides* species compared to germ‐free mice. A similar effect is seen for *C. difficile* that upregulates its sialic acid catabolism in mice colonised with wild‐type *Bacteroides thetaiotaomicron* compared to mice colonised with a sialidase‐deficient *Bacteroides* mutant (Ng et al., 2013).

Differences in host tropism of *C. jejuni* strains and host mucin composition underscore the importance of choosing the right intestinal epithelial model to investigate virulence of *C. jejuni fuc* + strains. In a piglet model of human disease, the NCTC 11168 *fuc* + strain has been shown to possess a competitive advantage when colonising the intestinal tract (Stahl et al., 2011). In our studies, we used a confluent monolayer of human intestinal Caco‐2 cells and observed that invasion of a *C. jejuni fuc +* strains was enhanced by fucosidase activity from a co‐culture with *B. fragilis*. In chicken, *C. jejuni* colonises the intestinal tract as a commensal and hence does not invade. Chicken mucins have an inhibitory effect on *C. jejuni* invasion into epithelial cells and, in these animals, *fuc* + strains do not have a competitive advantage over other *C. jejuni* strains. However, when chickens were fed additional l‐fucose, the *C. jejuni fuc* + wild‐type strain was more effective in colonising the intestinal tract compared to a fucose permease knockout strain (Byrne, Clyne, & Bourke, 2007; Stahl et al., 2011). Compared to human mucins, chicken mucins contain a large amount of sulfate modifications on their mucin *O*‐glycans (Struwe et al., 2015). One hypothesis is that the sulfate modifications block the function of exogenous fucosidases (Roberton & Wright, 1997). This hypothesis is supported by a mouse model where a decrease in sulfation enhanced intestinal penetrability by pathogens, including *C. jejuni* (Dawson et al., 2009). Interestingly, the occurrence of inflammatory bowel disease in humans, which is characterised by decreased mucus barrier function, has been correlated to an altered microbiota with an increased sulfate‐reducing bacterial population (Ijssennagger, van der Meer, & van Mil, 2016). Therefore, differences in mucin *O*‐glycan sulfation and surrounding microbiota could contribute to *C. jejuni fuc +* host tropism. The accessibility of terminal l‐fucosides on mucin *O*‐glycans for bacterial fucosidases in the presence or absence of specific sulfation patterns is, therefore, an interesting area for future studies.

*C. jejuni* is the leading cause of human bacterial gastroenteritis, but our understanding of *C. jejuni* pathogenicity is limited. Our work shows that release of l‐fucose by secreted fucosidases from commensal bacteria is an important determinant in *C. jejuni fuc* + growth and invasion (Figure 6). The novel insight that *C. jejuni fuc* + strains are, in part, dependent on commensal fucosidases and that these enzymes can be targeted with tailor‐made inhibitors provide opportunities for further research. The development of potent and selective bacterial fucosidase probes and inhibitors could lead to novel diagnostic and intervention strategies to target hyperinvasive *C. jejuni fuc* + infections.

## 4 EXPERIMENTAL PROCEDURES

### 4.1 Bacterial strains and media

*Campylobacter jejuni* strains, NCTC 11168 (clinical strain), 129,108 (intestinal isolate from patient with recurrent infections), were used in this study (Endtz et al., 1993; Skirrow, 1977). The complete sequence of the *fuc* operon of *C. jejuni* 108 is deposited at GenBank (CP053854). Routine growth of *C. jejuni* was carried out on 5% saponin‐lysed horse blood agar plates (Biotrading) or in HI liquid medium, supplemented with chloramphenicol (20 μg/mL), and/or kanamycin (25 μg/mL) as needed. Cultures were incubated at 37°C in microaerobic incubators with a gas concentration of 80% N_2_, 10% CO_2_, 5% O_2_, 5% H_2_. *Bacteroides fragilis* (NTCT9343) and *Bacteroides thetaiotaomicron* (DSMZ 2079) were grown anaerobically at 37°C in basal medium (tryptone yeast extract glucose, TYG) (Bacic & Smith, 2008) or Mega medium (Wu et al., 2015).

### 4.2 Generation of *C. jejuni* deletion strains

A *C. jejuni* deletion mutant in the gene, *cj0486*, was constructed by insertion of a chloramphenicol resistance cassette into the *cj0486* open reading frame. The target genes were PCR‐amplified using the primers YL017_Fw (AGCAAGTTTGAGCATGATAG) and YL018_Rv (TCTTCTAAAGAAGCGCTAGC) with genomic DNA of strains NCTC 11168 and 108 as template. The PCR products were cloned into the pJET1.2 plasmid using the manufacturer's protocol. The deletion plasmids were created by blunt insertion of the *Sma*I‐cut chloramphenicol cassette from pAV35 into the *Spe*I‐cut pJET1.2*‐cj0486*. The knock‐out plasmids were transformed into the corresponding *C. jejuni 11*,*168* and 108 strains by electroporation and transformants were selected on the ability to grow on chloramphenicol, containing selective agar plates. *Campylobacter jejuni* deletion strains were confirmed by PCR analysis.

### 4.3 L‐Fucose growth assays

DMEM medium (GIBCO) was supplemented with 10 mM l‐fucose (Sigma‐Aldrich, Netherlands) and filter sterilised (0.2 μm pore size). Wild type and mutant strains were grown for 4 days on saponin agar plates supplemented with corresponding antibiotics at 42°C under microaerophilic conditions. Several colonies were picked and plated onto fresh saponin agar plate and grown for 24 hr at 37°C under microaerophilic conditions. One colony was picked and grown overnight in HI at 37°C. These cultures were used to inoculate the growth medium at an OD_600_ of 0.1. Aliquots of this cell suspension were pipetted into a 96‐well plate for use with a synergy HTX plate reader. The plates were placed inside the hypoxic glove box (10% CO_2_, 5% O_2,_ 85% N_2_) in a plate reader and incubated at 37°C with moderate, continuous shaking for 20 hr with OD measurements every 10 min. Each growth condition was assessed in triplo and three biological replicates were performed. Statistical analysis was performed using a Student *t* test.

### 4.4 4‐Umbelliferon fucopyranoside assay for fucosidase activity

The enzymatic activity of α‐l‐fucosidases was assayed at 37°C by incubation with 100 μM 4‐methylumbelliferyl‐α‐l‐fucopyranoside as substrate (Sigma‐Aldrich) in 150 mM McIlvain buffer, pH 4.5. The recombinant α‐(1–2,3,4,6)‐l‐Fucosidase (*Homo sapiens* fucosidase FucHS; Megazyme) was diluted 1:500 to determine its activity. To determine fucosidase activity in *B. fragilis or B. thetaiotaomicron* supernatant fractions, concentrated supernatant (40 times over 10 K MWCO spinfilters) of an anaerobic overnight bacterial culture at 37°C was mixed 1:1 with pH 4.5 McIlvain buffer. As a negative control, the samples were boiled at 95°C for 10 minutes. The reactions were quenched by adding excess bicarbonate buffer (pH 10), after which fluorescence was measured with a fluorimeter Fluorstar Omega (BMG Labtech) using λ_EX_ 366 nm and λ_EM_ 445 nm.

### 4.5 Quantification of fucosidase activity by in‐gel fluorescence

Recombinant α‐(1–2,3,4,6)‐l‐Fucosidase (FucHS; Megazyme) and secreted fucosidases of *B. fragillis* were prepared as described above followed by incubation with 2 μM of the fluorescent activity‐based probe, JJB256 (Jiang et al., 2015), for 30 min at 37°C. The samples were denatured with Laemmli buffer for 5 min at 95°C and run in the dark on a 10% SDS‐PAGE gel. The gels were scanned with an Amersham imager in the CY2 channel. Signal intensity was quantified using ImageJ software.

### 4.6 Mucin growth assays

Ten milligram of porcine gastric mucin (PGM; Sigma Aldrich) was suspended in 1 mL milliQ and UV‐killed four times at 100.000 μJoule in a Stratalinker (Stratagene). For fucosidase treatment of mucins with recombinant enzyme, 50 mU of α‐(1–2,3,4,6)‐L‐Fucosidase (FucHS; Megazyme;) was added to the 10 mg/ml mucin solution and incubated for 18 hr at 37°C. To harvest secreted fucosidases of *B. fragillis*, a 6 ml anaerobic overnight culture of *B. fragillis* grown in Mega medium at 37°C was concentrated 60 times using 10 K MWCO spinfilters (ThermoFisher Scientific). As a negative control, FNJ (CAS 99212–30‐3, Carbosynth) was added to the secreted fraction with a final concentration of 100 μM to inhibit fucosidase activity. The concentrated secreted fraction was added to the 10 mg/ml mucin aliquot in the ratio 1:8 and incubated for 18 h at 37°C. The 10 mg/ml mucin aliquots were diluted in DMEM medium to obtain a final concentration of 1 mg/ml treated or untreated mucins. *Campylobacter jejuni* 108 was prepared for growth assays as described above and added to the DMEM medium, containing treated or untreated mucins, at an OD_600_ of 0.01. Growth of *C. jejuni* was quantified by counting colony‐forming units (CFU) on saponin plates incubated at 37°C under microaerophilic conditions for 24 hr. Statistical analysis was performed using a Student *t* test.

### 4.7 Mammalian cells and culture conditions

The human gastrointestinal epithelial cell line, Caco‐2 (ATCC‐HTB‐37), was routinely cultured in 25 cm^2^ flasks in Dulbecco's modified Eagle's medium (DMEM), containing 10% fetal calf serum (FCS), at 37°C in 10% CO_2_. For *C. jejuni* gentamicin protection invasion assays, Caco‐2 cells were split into six‐well plates and grown for 5 days to form a monolayer before *C. jejuni* infection. For microscopy analysis, the cells were cultured on circular glass coverslips in 24‐well plates.

### 4.8 Immunofluorescence of fucosylation levels on intestinal epithelial cells

Caco‐2 cells were grown on coverslips as described above and fixed with 4% paraformaldehyde in PBS (Affimetrix) for 30 min at room temperature. Cells were washed twice with Dulbecco's Phosphate Buffered Saline (DPBS, D8537, Sigma Aldrich) before they were permeabilized in binding buffer with 0.1% saponin (Sigma Aldrich) and 0.2% BSA (Sigma Aldrich) in PBS for 30 minutes. Next, coverslips were incubated with the blood group antigen H (O) type 1 mouse monoclonal antibody biotin (Invitrogen 17–206) or UEA‐I conjugated with Texas red (EY laboratories T‐2201‐2) for 1 hr, followed by four washing steps with binding buffer. The coverslips were incubated with streptavidin‐488 (Thermo Fisher S11223) for 1 hr at room temperature. Coverslips were washed three times with PBS and once with MilliQ and embedded in Prolonged diamond mounting solution (Thermo Fisher Scientific). Images were collected on LEICA SPE‐II confocal microscope in combination with LEICA LAS AF software.

### 4.9 *Campylobacter jejuni* infection assays

*Campylobacter jejuni* cultures were grown in HI for 24 hr at 37°C under microaerophilic conditions and adjusted to OD_600_ of 0.05 in 1 mL DMEM medium. Five day‐grown Caco‐2 cells were washed twice with DMEM without FCS. Bacteria were added to the cells in DMEM without FCS (+/− 10 mM l‐fucose; +/− 1 μL FucHS) at MOI = 100 and incubated under microaerophilic conditions at 37°C for 3.5 hr. The cells were washed five times with DMEM without FCS and replaced with DMEM without FCS, containing 250 μg/ml gentamicin, and incubated for 3 hr at 37°C to kill extracellular bacteria. The cells were then washed three times with PBS and lysed with 0.5% Triton X‐100 in PBS for 5 min at 37°C. Serial dilutions were made and plated on saponin agar plates that contained appropriate antibiotics. Plates were incubated at 37°C under microaerophilic conditions and the number of colony‐forming units was (CFU) determined. Statistical analysis was performed using a Student *t* test.

### 4.10 *Bacteroides fragilis* and *C. jejuni* co‐culture infection assay

Caco‐2 cells and *C. jejuni* 108 were prepared as mentioned above, *B. fragilis* (OD_600_ 1.0 of overnight culture; MOI 10) was added before addition of *C. jejuni*. FNJ (CAS 99212–30‐3, Carbosynth) was added to the medium simultaneously with *B. fragilis* with a final concentration of 100 μM, to inhibit fucosidase activity. Invasion of *C. jejuni* was determined as described above.

## CONFLICT OF INTEREST

The authors declare no conflict of interest.

## AUTHOR CONTRIBUTION

Yvette Luijkx designed and performed the experiments, analysed the data, prepared the figures and wrote the manuscript. Jianbing Jiang and Herman S. Overkleeft synthesised the activity‐based fucosidase probe (JJB256). Nancy Bleumink and Marc Wösten assisted with experimental design. Karin Strijbis and Tom Wennekes designed the study, wrote and revised the manuscript. All authors read and approved the final manuscript.
